# Echocardiographic assessment for cardiopulmonary function in patients with congenital heart disease-related pulmonary arterial hypertension

**DOI:** 10.1186/s12890-024-03113-7

**Published:** 2024-06-28

**Authors:** Lifang Yang, Dongling Luo, Taoran Huang, Xiaoshan Li, Guolin Zhang, Caojin Zhang, Hongwen Fei

**Affiliations:** 1 Guangdong Cardiovascular Institute, Guangdong Provincial People’s Hospital (Guangdong Academy of Medical Sciences), Southern Medical University, 106 Zhongshan Er Road, Guangzhou, Guangdong Province 510100 China; 2 Shantou University Medical College, Shantou, Guangdong Province China; 3 Guangdong Medical University, Zhanjiang, Guangdong Province China

**Keywords:** Congenital heart disease-related pulmonary arterial hypertension, Cardiopulmonary exercise test, Echocardiography, Right ventricle-pulmonary arterial coupling

## Abstract

**Background:**

For patients with congenital heart disease-related pulmonary arterial hypertension (CHD-PAH), cardiopulmonary exercise testing (CPET) can reflect cardiopulmonary reserve function. However, CPET may not be readily accessible for patients with high-risk conditions or limited mobility due to disability. Echocardiography, on the other hand, serves as a widely available diagnostic tool for all CHD-PAH patients. This study was aimed to identify the parameters of echocardiography that could serve as indicators of cardiopulmonary function and exercise capacity.

**Methods:**

A cohort of 70 patients contributed a total of 110 paired echocardiogram and CPET results to this study, with 1 year interval for repeated examinations. Echocardiography and exercise testing were conducted following standardized procedures, and the data were collected together with clinically relevant indicators for subsequent statistical analysis. Demographic comparisons were performed using t-tests and chi-square tests. Univariate and multivariate analyses were conducted to identify potential predictors of peak oxygen uptake (peak VO_2_) and the carbon dioxide ventilation equivalent slope (VE/VCO_2_ slope). Receiver operating characteristic (ROC) analysis was used to assess the performance of the parameters.

**Results:**

The ratio of tricuspid annular plane systolic excursion to pulmonary artery systolic pressure (TAPSE/PASP) was found to be the only independent indicator significantly associated with both peak VO_2_ and VE/VCO_2_ slope (both *p* < 0.05). Additionally, left ventricular ejection fraction (LVEF) and right ventricular fractional area change (FAC) were independently correlated with the VE/VCO_2_ slope (both *p* < 0.05). TAPSE/PASP showed the highest area under the ROC curve (AUC) for predicting both a peak VO_2_ ≤ 15 mL/kg/min and a VE/VCO_2_ slope ≥ 36 (AUC = 0.91, AUC = 0.90, respectively). The sensitivity and specificity of TAPSE/PASP at the optimal threshold exceeded 0.85 for both parameters.

**Conclusions:**

TAPSE/PASP may be a feasible echocardiographic indicator for evaluating exercise tolerance.

## Background

Congenital heart disease-related pulmonary arterial hypertension (CHD-PAH) is a life-threatening chronic condition typically arising from intracardiac or extracardiac shunts, leading to both volume and pressure overload. It often results in diminished exercise tolerance [1, 2]. Regular assessment of activity levels is recommended for individuals with CHD-PAH [1]. Cardiopulmonary exercise test (CPET) is a noninvasive procedure capable of objectively assessing cardiopulmonary reserve function and exercise tolerance. It offers valuable insights into exercise capacity, gas exchange dynamics during exercise, ventilator efficiency, and cardiac function. Besides, CPET also aids in developing exercise regimens and holds prognostic value for CHD-PAH patients [3–8]. Early studies have identified peak oxygen uptake (peak VO_2_) and the carbon dioxide ventilation equivalent slope (VE/VCO_2_ slope) from CPET as univariate biomarkers with prognostic implications in this population [6, 9]. In addition, according to the European Society of Cardiology and the European Respiratory Society (ESC/ERS) guidelines for risk stratification in PAH patients, a peak VO_2_ ≤ 15 ml/kg/min or VE/VCO_2_ slope ≥ 36 is considered an intermediate-high risk determinant for prognosis [10]. However, CPET may not be feasible for all PAH patients, particularly those with contraindications or limited mobility due to disability. Hence, there is a pressing need for safer and simpler diagnostic modalities to assess the severity and exercise capacity of PAH patients.

Echocardiography, known for its safety, simplicity, and cost-effectiveness, is recommended as the primary screening tool for initial assessment and follow-up in patients with CHD-PAH [1, 10]. Several echocardiographic parameters have been identified to correlate with exercise capacity and demonstrate prognostic significance in PAH patients [11–13].

In view of these findings, our aim was to identify an echocardiographic parameter capable of reflecting exercise capacity in patients with CHD-PAH.

## Methods

### Patients

The study included patients who met the following criteria from October 2021 to April 2023 at Guangdong Provincial People’s Hospital. Inclusion criteria comprised individuals who were (1) aged ≥ 18 years, (2) diagnosed with hemodynamic criteria consistent with PAH as per catheterization, including mean pulmonary arterial pressure > 20 mmHg, pulmonary arterial wedge pressure ≤ 15 mmHg, and pulmonary vascular resistance > 2 Wood units, in accordance with the 2022 ESC/ERS pulmonary hypertension guidelines, and clinically classified as having CHD-PAH, and (3) underwent echocardiography, 6-minute walk distance (6MWD), and CPET within 48 h. Patients with any of the following conditions were excluded: (1) other forms of pulmonary hypertension, (2) refusal to provide informed consent. After one year of enrollment, patients were recalled for repeat examinations.

### Echocardiography

Routine echocardiographic measurements were performed with patients positioned in the left lateral decubitus position using commercially available echocardiography systems (Philips EPIQ 7 C, Philips CV x, or Philips iE Elite). Images from three consecutive beats were digitally stored for offline analysis (QLAB 13.0, Philips Andover, MA). Various parameters including left ventricular ejection fraction (LVEF), right atrial area (RAA), tricuspid annular plane systolic excursion (TAPSE), right ventricular free wall thickness (RVWT), peak systolic velocity of the tricuspid annulus (S’), right ventricular fractional area change (FAC), echocardiography-estimated pulmonary arterial mean pressure (ePAMP), and echocardiography-estimated pulmonary arterial systolic pressure (ePASP) were measured based on current guidelines [14]. Among them, ePASP was calculated using the tricuspid regurgitation jet velocity, combined with an estimate of right atrial pressure derived from inferior vena cava diameter and respiratory changes. Echocardiography-estimated pulmonary arterial diastolic pressure (ePADP) was estimated from the velocity of the end-diastolic pulmonary regurgitant jet, also combined with the estimated right atrial pressure. The formula for calculating ePAMP is: ePAMP = 1/3(ePASP) + 2/3(ePADP). Additionally, right atrial and right ventricular two-dimensional speckle tracking imaging parameters were analyzed. Images of 4-chamber apical views focused on the right ventricle were obtained with a sector narrowing of 30°-60° and an acquisition frequency of 60–90 images per second [15]. Right atrial strain during the reservoir phase (RASr) and right ventricular free wall longitudinal strain (RVFWLS) were included in the study.

### Cardiopulmonary exercise testing

CPET was performed using the Alfred Schiller AG CS-200 cardiopulmonary exercise testing system. Following strict multistage calibration, patients underwent symptom-limiting maximal exercise tests on a bicycle ergometer (Ergoselect, Ergoline 900, Germany), with SpO_2_ monitored using a fingertip pulse oximeter (Heal Force, PC-60C1, China). The Ramp protocol was adopted, and patients were encouraged to exert maximal effort in the absence of discomfort. Expected values for each parameter were referenced from the Wasserman formula. Recorded CPET parameters included peak oxygen uptake (peakVO_2_), carbon dioxide output (VCO_2_), minute ventilation (VE), and VE/VCO_2_ slope. The anaerobic threshold was determined using the V-slope method.

### Six-minute walk test

All patients underwent a non-encouraged 6-minute walk test in a 30-meter-long corridor under the same environmental conditions. Throughout the test, participants’ heart rate and oxygen saturation were monitored every minute, with blood pressure measured at the beginning and end of the test. Upon completion of the 6 min, the walk distance was recorded, and participants completed the Borg Dyspnea and Fatigue Scale questionnaires.

### Statistical analysis

Data analysis was conducted using EmpowerStats (www.empowerstats.com, X&Y Solutions, Inc., Boston, MA) and R software version 4.1.1 (http://www.r-project.org). Continuous variables following a normal distribution were expressed as mean ± standard deviation, while categorical variables were expressed as numbers and proportions. Chi-square tests were used for group comparisons of categorical variables. Student’s *t*-*tests* were used for normally distributed continuous variables. Kruskal‒Wallis test was applied for skewed continuous variables. Indicators with a *p* value < 0.05 were included in multivariate analysis, progressively eliminated via forward stepwise regression. Variables with a *p* value < 0.05 in the multivariate analysis were incorporated into receiver operating characteristic (ROC) curve analysis, and the area under the curve (AUC) was calculated. The optimal predictive threshold was determined, and sensitivity and specificity of each index for predicting intermediate-high risk, as stratified by CPET according to the 2022 ESC/ERS pulmonary hypertension guidelines, were calculated.

## Results

### Baseline characteristics

A total of 70 patients were diagnosed with CHD-PAH, among whom 40 underwent two sets of echocardiography and CPET within a one-year interval. Finally, they contributed 110 paired echocardiogram and CPET results. Baseline characteristics of the overall population are presented in Table 1. Patients had a mean age of 35 ± 8 years, with the majority of female (87%), and the majority falling into World Health Organization functional class (WHO-FC) I-II (96%). Only the distributions of WHO-FC were significantly different between the subgroups (both *p* < 0.01).

Compared to individuals with a peak VO_2_ > 15 ml/kg/min, those with a peak VO_2_ ≤ 15 ml/kg/min demonstrated lower SpO_2_ and shorter 6MWD (*p* = 0.001, *p* < 0.001, respectively). Similarly, patients with a VE/VCO_2_ slope ≥ 36 exhibited lower SpO_2_ and shorter 6MWD (*p* < 0.001, *p* < 0.001, respectively). Within the subgroup with a peak VO_2_ ≤ 15 ml/kg/min, RAA, RVWT, S’, ePASP, and RVFWLS were higher (all *p* < 0.001), while FAC, TAPSE/PASP, and RASr were lower (all *p* < 0.001). Conversely, in the subgroup with a VE/VCO_2_ slope ≥ 36, RAA, RVWT, ePAMP, ePASP, and RVFWLS were higher (all *p* < 0.01), while FAC and TAPSE/PASP were lower (both *p* < 0.001).

**Table 1:** Baseline characteristics of study population

Variables	Total		Peak VO_2_ (ml/kg/min)		*p*-value	VE/VCO_2_ slope		*p*-value
	*n* = 110		> 15 *n* = 48	≤ 15 *n* = 62		< 36 *n* = 45	≥ 36 *n* = 64	
**Demographic characteristics**								
Age (years)	35 ± 8		34 ± 8	35 ± 8	0.389	33 ± 6	35 ± 9	0.225
Sex					0.950			0.106
Female	96 (87%)		42 (88%)	54 (87%)		42 (93%)	53 (83%)	
Male	14 (13%)		6 (12%)	8 (13%)		3 (7%)	11 (17%)	
BMI (kg/m^2^)	19.5 ± 3.2		18.9 ± 2.8	20.0 ± 3.4	0.059	20.0 ± 3.0	19.1 ± 3.3	0.140
WHO-FC					< 0.001			0.005
I	40 (37%)		28 (58%)	12 (20%)		24 (55%)	16 (26%)	
II	63 (59%)		19 (40%)	44 (75%)		20 (45%)	42 (68%)	
III	4 (4%)		1 (2%)	3 (5%)		0 (0%)	4 (6%)	
**Clinical parameters**								
Heart rate (b.p.m)	88 ± 13		85 ± 13	91 ± 12	0.014	84 ± 13	91 ± 12	0.007
SpO_2_ (%)	94 ± 5		95 ± 5	92 ± 5	0.001	97 ± 3	91 ± 5	< 0.001
6MWD (m)	473 ± 79		514 ± 69	444 ± 73	< 0.001	510 ± 68	444 ± 73	< 0.001
**Echocardiography characteristics**								
LVEF (%)	69 ± 6		68 ± 5	68 ± 7	0.918	69 ± 5	68 ± 7	0.355
RAA (cm^2^)	16.5 ± 5.9		14.1 ± 4.8	18.4 ± 6.0	< 0.001	14.3 ± 4.7	18.0 ± 6.2	0.001
TAPSE (mm)	17.7 ± 4.3		18.5 ± 5.5	17.0 ± 3.0	0.062	18.3 ± 5.0	17.2 ± 3.9	0.200
RVWT (mm)	7.9 ± 3.0		6.7 ± 2.7	8.8 ± 2.9	< 0.001	6.0 ± 2.4	9.2 ± 2.6	< 0.001
S’ (cm/s)	11.3 ± 2.6		12.3 ± 2.4	10.5 ± 2.5	< 0.001	11.6 ± 2.6	11.1 ± 2.6	0.264
FAC (%)	37 ± 9		41 ± 7	35 ± 9	< 0.001	41 ± 6	35 ± 9	< 0.001
ePAMP (mmHg)	46 ± 20		41 ± 22	50 ± 19	0.065	32 ± 15	55 ± 19	< 0.001
ePASP (mmHg)	80 ± 33		54 ± 22	98 ± 28	< 0.001	53 ± 20	100 ± 28	< 0.001
TAPSE/PASP (mm/mmHg)	0.28 ± 0.18		0.41 ± 0.20	0.19 ± 0.08	< 0.001	0.40 ± 0.19	0.20 ± 0.11	< 0.001
RASr	36.6 ± 14.7		42.8 ± 13.6	31.8 ± 13.8	< 0.001	38.6 ± 14.1	35.1 ± 15.2	0.234
RVFWLS	-20.7 ± 6.4		-23.4 ± 5.5	-18.6 ± 6.3	< 0.001	-22.8 ± 5.9	-19.2 ± 6.5	0.005

Data: mean ± standard deviation or n (%)

Abbreviations: M, male; F, female; BMI, body mass index; WHO-FC: World Health Organization functional class; CHD, congenital heart disease; 6MWD, 6-minute walk distance; LVEF, left ventricular ejection fraction; RAA, right atrial area; TAPSE, tricuspid annular plane systolic excursion; RVWT, right ventricular wall thickness; S’, tricuspid valve annulus peak systolic velocity; FAC, fractional area change; ePAMP, echocardiography estimated mean pulmonary arterial pressure; ePASP, echocardiography estimated systolic pulmonary arterial pressure; RASr, right atrial strain during the reservoir phase; RVFWLS, right ventricular free wall longitudinal strain

### Correlation analysis

**Fig. 1:**
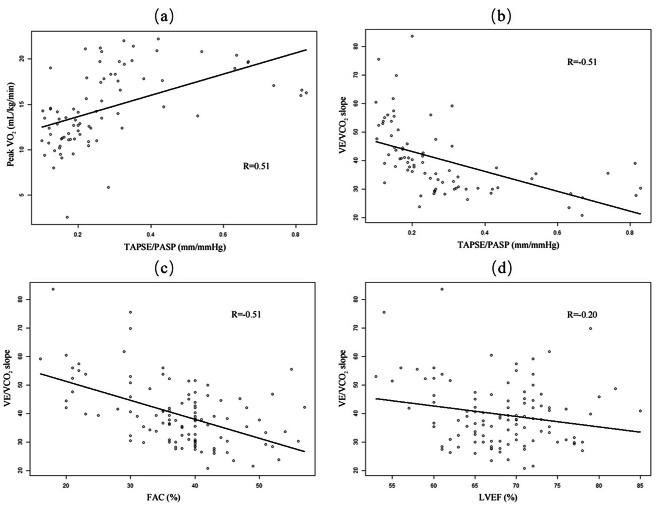
Linear correlation analysis between echocardiography parameters and the main results of the cardiopulmonary exercise testing (**a**) Relationship between peak VO_2_ and TAPSE/PASP. (**b**) Relationship between VE/VCO_2_ slope and TAPSE/PASP. (**c**) Relationship between VE/VCO_2_ slope and FAC. (**d**) Relationship between VE/VCO_2_ slope and LVEF. Abbreviations: TAPSE/PASP, the ratio of tricuspid annular plane systolic excursion and systolic pulmonary arterial pressure; FAC, fractional area change; LVEF, left ventricular ejection fraction

The results of univariate and multivariate analyses of the predictive factors for peak VO_2_ and VE/VCO_2_ slope are presented in Tables 2 and 3, respectively. Figure 1 depicts the linear correlation analysis between the echocardiographic parameters and the main CPET results in the overall population. According to our multivariate analysis, only TAPSE/PASP was independently associated with peak VO_2_ (*β* = 5.72, *p* = 0.019). In the multivariate analysis of the VE/VCO_2_ slope, LVEF, FAC, and TAPSE/PASP were found to be independently predictive factors (*β* = -0.48, *p* = 0.022; *β* = -0.65, *p* < 0.001; *β* = -23.29, *p* = 0.024, respectively). LVEF weakly correlated with VE/VCO_2_ slope, with the correlation coefficient of 0.20. Among the echocardiographic parameters assessed in our study, only TAPSE/PASP demonstrated independent and moderate linear correlation with both peak VO_2_ and VE/VCO_2_ slope.

**Table 2:** Univariate and multivariate analyses of echocardiographic and clinical variables associated with peak VO_2_

Exposure	univariate analysis			multivariate analysis	
	β(95% CI)	*p*		β(95% CI)	*p*
**Age**	-0.06 (-0.15, 0.02)	0.165		-	-
**Sex**					
**Female**	0			-	
**Male**	-0.27 (-2.42, 1.87)	0.803		-	-
**LVEF**	0.05 (-0.07, 0.17)	0.442		-	-
**RAA**	-0.23 (-0.34, -0.11)	0.000		-	-
**RVWT**	-0.60 (-0.82, -0.39)	< 0.001		-	-
**S’**	0.44 (0.18, 0.71)	0.001		-	-
**FAC**	0.15 (0.07, 0.23)	< 0.001		-	-
**TAPSE/PASP**	11.76 (7.36, 16.16)	< 0.001		**5.72 (0.61, 10.84)**	**0.019**
**RASr**	0.08 (0.03, 0.13)	0.001		**-**	**-**
**RVFWLS**	-0.18 (-0.29, -0.08)	0.001		**-**	**-**
**6MWD**	0.02 (0.01, 0.03)	< 0.001		**-**	**-**

Abbreviations: LVEF, left ventricular ejection fraction; RAA, right atrial area; RVWT, right ventricular wall thickness; S’, tricuspid valve annulus peak systolic velocity; FAC, fractional area change; TAPSE/PASP, the ratio of tricuspid annular plane systolic excursion and systolic pulmonary arterial pressure; RASr, right atrial strain during the reservoir phase; RVFWLS, right ventricular free wall longitudinal strain; 6MWD, 6-minute walk distance

**Table 3:** Univariate and multivariate analyses of echocardiographic and clinical variables associated with the VE/VCO_2_ slope

Exposure	Univariate analysis			Multivariate analysis	
	β(95% CI)	*p*		β(95% CI)	*p*
**Age**	0.06 (-0.20, 0.33)	0.644		-	-
**Sex**					
**Female**	0			-	
**Male**	1.71 (-4.63, 8.05)	0.599		**-**	-
**LVEF**	-0.37 (-0.71, -0.02)	0.040		**-0.48 (-0.87, -0.08)**	**0.022**
**RAA**	0.50 (0.15, 0.85)	0.007		**-**	**-**
**RVWT**	1.64 (1.00, 2.29)	< 0.001		**-**	**-**
**S’**	-0.91 (-1.72, -0.11)	0.029		**-**	**-**
**FAC**	-0.66 (-0.87, -0.45)	< 0.001		**-0.65 (-0.98, -0.32)**	**< 0.001**
**TAPSE/PASP**	-34.82 (-47.70, -21.94)	< 0.001		**-23.29 (-43.02, -3.56)**	**0.024**
**RASr**	-0.08 (-0.23, 0.07)	0.313		**-**	**-**
**RVFWLS**	0.61 (0.29, 0.92)	< 0.001		**-**	**-**
**6MWD**	-0.06 (-0.09, -0.03)	< 0.001		**-**	**-**

Abbreviations: LVEF, left ventricular ejection fraction; RAA, right atrial area; RVWT, right ventricular wall thickness; S’, tricuspid valve annulus peak systolic velocity; FAC, fractional area change; TAPSE/PASP, the ratio of tricuspid annular plane systolic excursion and systolic pulmonary arterial pressure; RASr, right atrial strain during the reservoir phase; RVFWLS, right ventricular free wall longitudinal strain; 6MWD, 6-minute walk distance

### ROC analysis

**Fig. 2:**
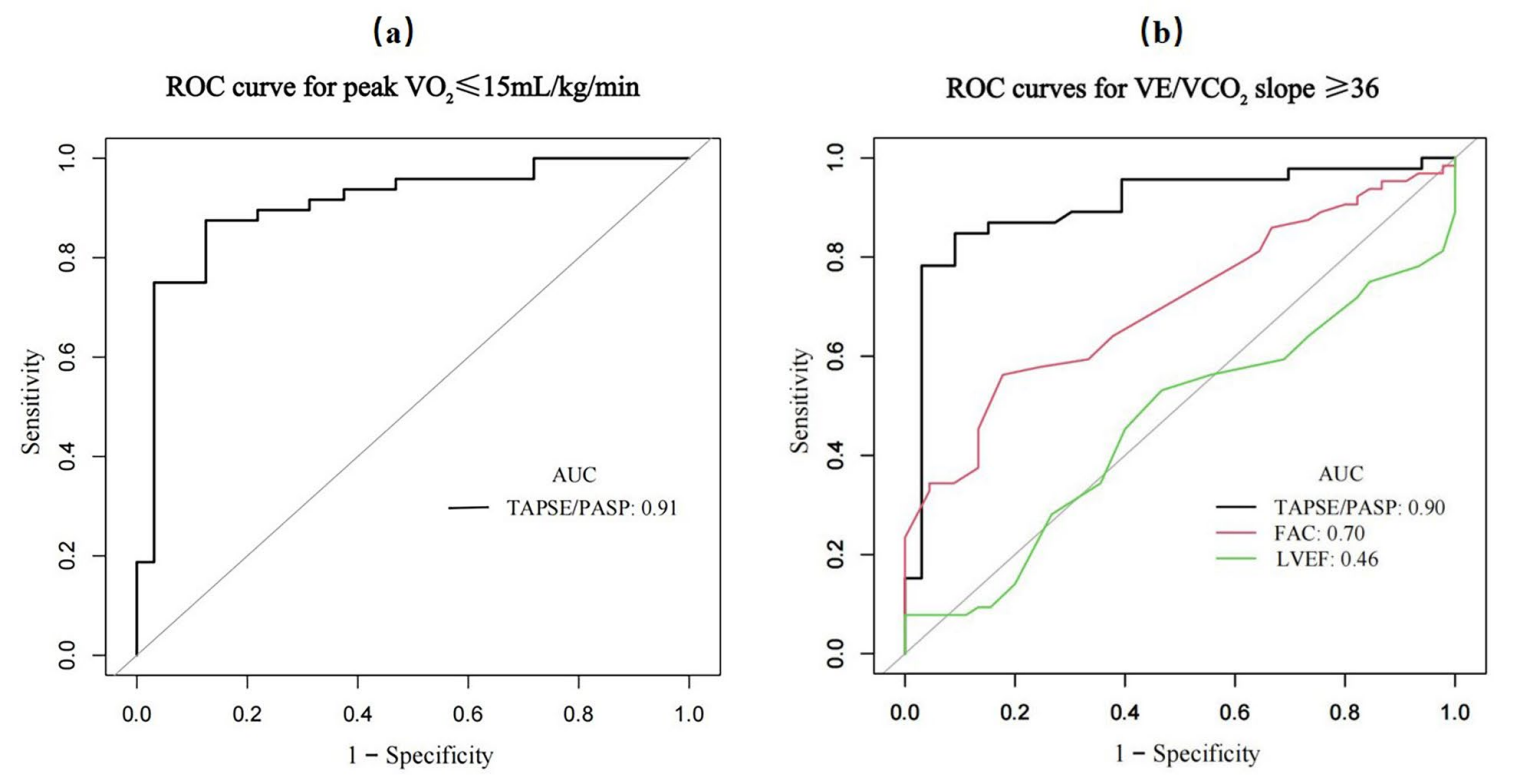
Receiver Operating Characteristic curves for predicting peak VO_2_ ≤ 15 mL/kg/min and VE/VCO_2_ slope ≥ 36. (**a**) ROC curve depicting the performance of TAPSE/PASP in predicting peak VO_2_ ≤ 15 mL/kg/min. (**b**) ROC curves illustrating the predictive capabilities of TAPSE/PASP, FAC, and LVEF for VE/VCO_2_ slope ≥ 36. Abbreviations: TAPSE/PASP, the ratio of tricuspid annular plane systolic excursion and systolic pulmonary arterial pressure; FAC, fractional area change

Figure 2; Table 4 shows the results of the ROC analysis for the overall population regarding the selected parameters in relation to peak VO_2_ and VE/VCO_2_ slope values. TAPSE/PASP exhibited the highest AUC for predicting both a peak VO_2_ result of ≤ 15 mL/kg/min and a VE/VCO_2_ slope result of ≥ 36 (AUC = 0.91, AUC = 0.90, respectively). Additionally, the optimal threshold for TAPSE/PASP to predict a peak VO_2_ result of ≤ 15 mL/kg/min was determined to be 0.26 mm/mmHg, with a sensitivity and specificity of 0.88 and 0.88, respectively. Similarly, to predict a VE/VCO_2_ slope ≥ 36, the cutoff for TAPSE/PASP was 0.23 mm/mmHg, with a sensitivity of 0.85 and specificity of 0.91. The AUC for the other two parameters, LVEF and FAC, which were independently associated with VE/VCO_2_ slope, were 0.46 and 0.70, respectively.

**Table 4:** Results of receiver operating characteristic curves for predicting peak VO_2_ ≤ 15 mL/kg/min and VE/VCO_2_ slope ≥ 36

Variables	AUC	95%CI	Best threshold	Specificity	Sensitivity
***peak VO***_***2***_ ≤ ***15 mL/kg/min***					
TAPSE/PASP	0.91	0.84–0.98	0.26	0.88	0.88
***VE/VCO***_***2***_ ***slope ≥ 36***					
TAPSE/PASP	0.90	0.83–0.98	0.23	0.91	0.85
FAC	0.70	0.61–0.80	36.5	0.82	0.56
LVEF	0.46	0.35–0.57	78.5	1.00	0.08

Abbreviations: AUC, area under the curve; TAPSE/PASP, the ratio of tricuspid annular plane systolic excursion and systolic pulmonary arterial pressure; FAC, fractional area change; LVEF, left ventricular ejection fraction

## Discussion

The findings of our study revealed a significant correlation between TAPSE/PASP and exercise capacity, as indicated by peak VO_2_ and VE/VCO_2_ slope during CPET in patients with PAH. TAPSE/PASP exhibited excellent performance, with higher AUC, sensitivity, and specificity in distinguishing intermediate-high risk patients, as stratified by CPET according to ESC/ERS pulmonary hypertension guidelines. To the best of our knowledge, this is the first study to compare the relationship between TAPSE/PASP and CPET in a PAH population.

Previous studies have suggested that various echocardiographic parameters, including right ventricular global longitudinal strain, RVFWLS, FAC, TAPSE, S’, and RAA, could classify PAH patients based on peak VO_2_ or VE/VCO_2_ slope [13]. Besides, Sljivic et al. have also demonstrated that the right ventricular global longitudinal strain and 3-dimensional right ventricle ejection fraction are strongly associated with exercise capacity in patients with heart failure and a reduced ejection fraction (HFrEF) [16]. Additionally, Liu et al. reported that right ventricular peak systolic strain might aid in classifying PAH patients according to exercise testing risk stratification cut-offs [12]. These studies suggest that right ventricular function parameters obtained via echocardiography may reflect cardiopulmonary reserve function and exercise capacity. However, our study revealed that among several commonly used echocardiographic parameters of right ventricular function, only TAPSE/PASP was independently and concurrently associated with both peak VO_2_ and VE/VCO_2_ slope after multivariate analysis. While FAC was moderately correlated with VE/VCO_2_ slope and demonstrated some ability to distinguish CHD-PAH patients at intermediate-high risk, LVEF exhibited weak correlation and poor classification ability, with an AUC of only 0.46 in the overall population. This aligns with previous findings suggesting that VE/VCO_2_ slope was strongly related to right ventricular function but poorly and even not related to left ventricular function [17–19].

As a direct indicator of exercise ability, 6MWD appeared to be less correlated with CPET than TAPSE/PASP. This could be attributed to the influence of various factors, including objective factors such as sex, age, height, weight, comorbidities, as well as subjective factors such as the learning curve and motivation [10].

Two-dimensional speckle tracking echocardiographic parameters, such as RASr and RVFWLS, did not emerge as independent influencing factors in our study. One potential explanation could be their linear correlation with TAPSE/PASP, which in turn exhibited a robust correlation with peak VO_2_ and VE/VCO_2_ slope.

Furthermore, the ROC analysis results in our study, including AUC, sensitivity, and specificity, highlighted the superiority of TAPSE/PASP over other parameters in predicting both peak VO_2_ ≤ 15 mL/kg/min and VE/VCO_2_ slope ≥ 36.

In patients with CHD-PAH, inadequate control of volume and pressure overload can lead to three-layer remodeling of distal precapillary pulmonary vessels, including uncontrolled growth of endothelial cells, smooth muscle cells, and fibroblasts, as well as infiltration of inflammatory cells, [20, 21] accompanied by an increase in pulmonary vascular resistance, resulting in right heart dysfunction and right ventricular-pulmonary artery uncoupling [22]. Damage to the pulmonary vascular bed can cause pulmonary ventilation-perfusion mismatch, manifested as an elevated VE/VCO_2_ slope in CPET. [23], while decreased exercise tolerance due to right heart dysfunction is reflected in a reduced peak VO_2_ [23]. Therefore, a right ventricle-pulmonary arterial coupling index may be associated with ventilatory inefficiency and maximal oxygen uptake.

TAPSE/PASP, initially proposed by Guazzi et al. in patients with left heart disease and Group 2 PH, [24] has been the most widely recognized and recommended noninvasive right ventricle-pulmonary arterial coupling index currently [10, 25, 26]. However, limited research has evaluated the correlation between this parameter and cardiopulmonary exercise ability. Legris et al. suggested a strong association between TAPSE/PASP and peak VO_2_ in patients with HFrEF [18]. In our study, TAPSE/PASP demonstrated a strong correlation with CPET values, reflecting cardiopulmonary function and exercise capacity, and efficiently classified patients at intermediate-high risk based on CPET parameters of ESC/ERS risk stratification. These findings suggest that TAPSE/PASP may provide additional information about exercise tolerance in disease assessment, clinical treatment, and follow-up for CHD-PAH patients, especially for high-risk patients unable to complete CPET.

This study had several limitations. It was conducted in a single center and requires validation in additional locations. The majority of patients in our study were WHO class I-II, limiting the generalizability of the results to patients with WHO class III-IV. Besides, patients with respiratory diseases that may impair ventilation reserve function were not excluded, potentially affecting CPET results. Additionally, longitudinal data were not included in this study, warranting further exploration of the consistency between TAPSE/PASP and changes in cardiopulmonary exercise.

## Conclusions

TAPSE/PASP were strongly associated with exercise capacity and exhibited the best classification ability, as evidenced by the highest AUC, sensitivity, and specificity, making it effective in distinguishing PAH patients with impaired aerobic performance and heightened risk stratification. Therefore, TAPSE/PASP holds considerable potential for facilitating a convenient and safe evaluation of exercise tolerance in patients with CHD-PAH.

## Data Availability

The datasets used and analysed during the current study are available from the corresponding author on reasonable request.

## Abbreviations

CHD-PAH: Congenital heart disease-related pulmonary arterial hypertension
CPET: Cardiopulmonary exercise test
peak VO_2_: Peak oxygen uptake
VE/VCO_2_ slope: Carbon dioxide ventilation equivalent slope
ESC/ERS: European Society of Cardiology and the European Respiratory Society
PAH: Pulmonary arterial hypertension
6MWD: 6-minute walk distance
LVEF: Left ventricular ejection fraction
RAA: Right atrial area
TAPSE: Tricuspid annular plane systolic excursion
RVWT: Right ventricular free wall thickness
S’: Peak systolic velocity of the tricuspid annulus
FAC: Fractional area change
ePAMP: Echocardiography-estimated pulmonary arterial mean pressure
ePASP: Echocardiography-estimated pulmonary arterial systolic pressure
PADP: Pulmonary arterial diastolic pressure
RASr: Right atrial strain during the reservoir phase
RVFWLS: Right ventricular free wall longitudinal strain
peakVO_2_: Peak oxygen uptake
VCO_2_: Carbon dioxide output
VE: Minute ventilation
VE/VCO_2_ slope: The carbon dioxide ventilation equivalent slope
ROC: Receiver operating characteristic
AUC: Area under the receiver operating characteristic curve
WHO-FC: World Health Organization functional class
TAPSE/PASP: The ratio of tricuspid annular plane systolic excursion to pulmonary artery systolic pressure
HFrEF: Heart failure and reduced ejection fraction
